# Prevalence of intestinal parasite, *Shigella* and *Salmonella* species among diarrheal children in Jimma health center, Jimma southwest Ethiopia: a cross sectional study

**DOI:** 10.1186/1476-0711-13-10

**Published:** 2014-02-05

**Authors:** Getenet Beyene, Haimanot Tasew

**Affiliations:** 1 Department of Laboratory Sciences and Pathology, College of Public Health and Medical Sciences, Jimma University, Jimma, Ethiopia

**Keywords:** Intestinal parasite, *Shigella*, *Salmonella*, Susceptibility test, Jimma, Ethiopia

## Abstract

**Background:**

Diarrheal disease continues to be an important cause of morbidity and mortality among young children in developing countries including Ethiopia. Globally, intestinal parasite, *Shigella* and *Salmonella* species remain major contributors to acute enteric infections. The study was aimed at determining the frequency of intestinal parasite, *Shigella* and *Salmonell*a species identified from diarrheic children at Jimma Health Centre, Jimma south west Ethiopia.

**Methods:**

A health institution based cross sectional study was conducted from March to November 2012. A structured questionnaire was used for collection of data on socio- demographic characteristics. Parasite and bacteria identification as well as susceptibility testing was done using standard parasitological and bacteriological procedures.

**Results:**

A total of 260 diarrheal children were included in the study. A total of 129 (49.6%) samples were positive for intestinal parasite, *Shigella* and *Salmonella* species. Of these, 107 (41.1%), 6 (2.3%) and 16 (6.2%) samples were positive for intestinal parasite, *Shigella* and *Salmonella* species respectively. The dominant isolated parasite was *G. lamblia* with prevalence of 13.5% followed by *A. lumbricoides* (11.5%). The least identified parasites were *Schistosoma mansoni and Taenia species* accounting 0.4% each. Multiple parasitic infections were observed in 19 (7.3%) patients. *Shigella* species showed hundred percent resistances to ampicillin, amoxacillin, and cotrimoxazole. All *Salmonella* isolates were resistant against amoxicillin. All *Shigella* and *Salmonella* species were susceptible to ceftriaxone, ciprofloxacin and gentamycin.

**Conclusion:**

The presence of reasonably high amount of intestinal parasite and *Salmonella* and *Shigella* species that are drug resistance to the commonly prescribed drugs is a treat to the children and community at large. Therefore, measures including health education, improvement of safe water supply, sanitation facilities and continuous monitoring of microbiological and antimicrobial surveillance is crucial.

## Introduction

Diarrheal disease continues to be an important cause of morbidity and mortality among young children in developing countries [1]. Children and young adults are the most affected, particularly in regions with limited resources and where hygienic measures are inadequate. Causes of diarrhea in endemic areas include a wide variety of bacteria, viruses, and parasites [2].

Gastrointestinal parasitic infections are amongst the most common infections worldwide. These cases are attributed to three common intestinal parasites: *Ascaris lumbricoides*, hookworm, and *Trichuris trichiura*. The global prevalence of parasitic diseases is estimated to be 478 million children for *A. lumbricoides*; 280 million for hookworms and 347 million children for *T. trichiura*[3]. In Ethiopia, intestinal parasitic infections are of serious public health concern [3]. According to a report by the Ministry of Health, helminthiasis is the third leading cause of outpatient visits in health institutions in 2005-2006 [4].

Recently, it was observed that the prevalence of intestinal parasitic infections in Jimma, Ethiopia [5] was as high as 86.2%; whereas in Tigray, the overall prevalence was 48.1% [6]. According to the study findings conducted in Angolela, Ethiopia, on school children by Nguyen *et al* (2012), one-third of the children were infected with protozoan while 7.1% were found to have helminthic infections [7].

Among the diarrhoeal pathogens, *Shigella* continues to play a major role in etiology of inflammatory diarrhoea and dysentery, thus presenting a serious challenge to public-health authorities worldwide [8]. The few studies conducted on shigellosis in Ethiopia revealed that, Shigellosis and the emergence of antimicrobial resistant *Shigella* species is a major health problem [9-14]. Recent data different health institutions in Ethiopia have indicated that salmonellosis is a common problem and also showed the presence of a number of serogroups/serotypes in humans, animals, food animals, food products animal origins and other food stuff [15-19].

Infections by most species of *Shigella* and *Salmonella* can be asymptomatic, or can be treated with rehydration solutions except for infection by invasive strains. The use of antibiotics might shorten the duration of diarrhea and limit the shedding of the organisms which otherwise might continue to spread among people and in to the environment and further pose a risk of transmission of infections. However, antimicrobial resistance is an overgrowing problem, and there is a need to monitor the susceptibility of common bacterial isolates to drugs used in the community to provide guidelines for the empirical treatment of bacterial infections.

In the present study, a prospective cross sectional study was conducted to determine the prevalence of intestine parasite, *Shigella* and *Salmonella* species among diahrroic children visiting Jimma Helath Center from March to November 2012, south West Ethiopia. We determined the prevalence of intestinal parasites, *Shigella* and *Salmonella* species in diarrhoeal stools of children including also the susceptibility to antimicrobial agents of the investigated pathogens.

## Material and methods

### Study design, area and period

A prospective cross sectional study was conducted to determine the prevalence of intestinal parasite, *Shigella* and *Salmonella* species among diarrheic children visiting Jimma Health Center, Jimma south West Ethiopia from March to November 2012.

### Sample size and sampling technique

A total of 260 children were participated as a study subject. The sample size was determined based on the prevalence rate of the study done by Mache on children at Jimma [15] and calculated with the formula recommended by Daniel W [20].

### Demographic data collection

Histories were taken from each child and informed consent was obtained from the parents or guardians before sample collection was attempted by the attending pediatrician. All relevant demographic, clinical and laboratory data were recorded and transferred to the questionnaire prepared for this study.

### Specimen collection and identification of pathogens

Freshly passed stool and rectal swab was collected, placed immediately in Cary Blair transport medium (Oxoid Ltd, Basingstoke, UK) and transported to the laboratory within six hours of collection. For identification of *Shigella* and *Salmonella* species, specimens were placed in Selenite F enrichment broth (Oxoid) and incubated at 37°C for 24 hours, then subcultured onto deoxycholate agar (DCA) and xylose lysine deoxycholate agar (XLD) (Oxoid) agar at 37°C for 18-24 hours. The growth of *Salmonella* and *Shigella* species was detected by their characteristic appearance on XLD agar (*Shigella*: red colonies, *Salmonella* red with a black centre) and DCA (*Shigella*: pale colonies, *Salmonella* black centre pale colonies). The suspected colonies were further tested through a series of biochemical tests to identify *Shigella* and *Salmonella* species [21].

### Parasitological examination of stool

Stool specimens were obtained from all participants and examined for the presence of intestinal parasite cysts, eggs, trophozoites and larvae. In the laboratory, slides were prepared directly for wet mount in saline as well as in iodine and then were microscopically examined initially under low power (10X10 magnifications) bright field then under high-power (40X40 magnification) bright field. Finally the sample was concentrated using the procedure of formalin ethyl acetate technique4 and iodine stained slides were prepared and examined microscopically.

### Susceptibility testing

Antimicrobial drug susceptibility testing was carried out using disk diffusion method according to Clinical Laboratory Standards Institute (CLSI) guide lines [22]. The antibiotic discs used and their concentrations were:-ceftriaxone (CRO, 30 μg), chloramphenicol (C, 30 μg), ciprofloxacin (CIP, 5 μg), gentamicin (GM, 10 μg), nalidixic acid (NA, 30 μg), trimethoprim-sulfamethoxazole/cotrimoxazole (SXT, 25 μg) ampicillin (AMP 10 μg) and amoxicillin (AML, 20 μg). All antibiotic were obtained from Oxoid Limited, Basingstoke Hampshire, UK. A standard inoculum adjusted to 0.5 McFarland was swabbed on to Muller- Hinton agar (Oxoid Ltd. Bashingstore Hampaire, UK); antibiotic disc were dispensed after drying the plate for 3-5 min and incubated at 37°C for 24 hours. The reference strains used as control were *E.coli* ATCC 25922.

Data were entered and analyzed using SPSS version 16.0 computer software. Ethical clearance was secured from Ethical Clearance Committee of College of Public Health and Medical Sciences Jimma University. Permission was obtained from Health center officials.

## Results

**Figure 1:**
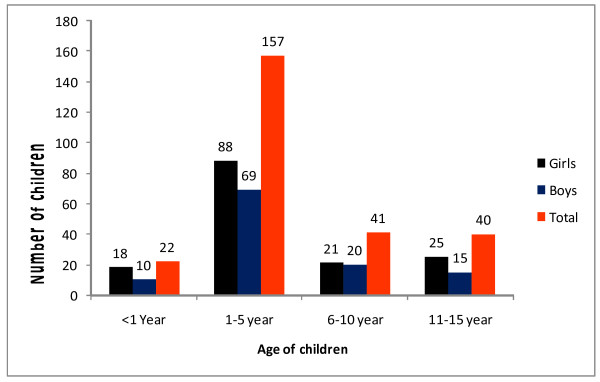
Age and sex distribution of the study group.

Stool specimens of 260 children were collected and examined for the presence of intestinal parasites and cultured for *Salmonella* and *Shigella* species. Out of the total 260 study participants 114 (43.8%) were males and 146 (56.2%) were females showing an overall male to female ratio 1:0.8. The age of the studied children ranges from one month of age to 15 years with mean age of five year (+SD 4.1): the majority (60.3%) of the study subjects were between 1-5 years of age and list frequency (8.4%) was observed for children less than one years old (Figure 1).

Out of the 260 stool samples, 129 (49.6%) samples were positive for intestinal parasite, *Shigella* and *Salmonella* species. Of these, 107 (41.1%), 6 (2.3%) and 16 (6.2%) samples were positive for intestinal parasite, *Shigella* and *Salmonella* species respectively. The dominant isolated parasite was *Giardia. lamblia* with prevalence of 13.5% followed by *A. lumbricoides* (11.5%). The least identified parasites were *Schistosoma mansoni and Taenia species* accounting 0.4% each (Table 1).

**Table 1:** Frequency of intestinal parasite and bacteria isolated from 260 diarrheic children

***Etiological pathogens***	***Patient*** **(*n*** **=** ***260*)** ***no*. (%)**
*Paraite*	
*A. lumbricoides*	30 (11.5)
*T. trichiura*	8 (3.1)
Hook worm	4 (0.4)
*H. nana*	20 (7.5)
*Taenia spp*	1 (0.4)
*S. mansoni*	1 (0.4)
*Giardia lamblia*	35 (13.5)
*E. histolytica*	8 (3.1)
***Total***	**107 (41.1)**
***Bacteria***	
*Shigella* species	6 (2.3)
*Salmonella* species	16 (6.2)
***Total***	**22 (8.5)**
***Enteropahogens total***	**129 (49.6)**

The distribution of enteropathogens according to the different age groups is listed in Table 2. The majority (58.1%) of enteropathogens were found in children aged 1-5 years. Whereas, 26 (20.2%), 21 (16.3%) and seven (5.4%) pathogens were observed in children within age groups of 6 – 10, 11 – 15 and less than one years, respectively. Multiple parasitic infections were observed in 19 patients (Table 3). Triple infection was observed in five children (1.5%) and double parasitic infections were observed among 14 children. The commonest parasites in multiple infections were *A. lumbricoides* + *G. lamblia*. The commonest double infections were *T. trichuria* + *G. lamblia* + *G. lamblia* + *E. histolytic*. The commonest triple infections were *A*. *lumbricoides* + *H. nana* + *G. lamblia* (15.8%). There was no confection of bacteria (*Salmonella* and *Shigella* species) and parasite among the children (Table 3).

**Table 2:** **Age distribution of intestinal parasite, *Shigella and Salmonella *** **species among the study participants**

	***No*. (%)**			
Age group in years	< 1	1–5	6–10	11–15
**Parasite**				
*A. lumbricoides*	-	13 (10.1)	10 (7.8)	7 (5.4)
*T. trichiura*	-	5 (3.9)	1 (0.8)	2 (1.6)
Hook worm	-	1 (0.8)	1 (0.8)	2 (1.6)
*H. nana*	-	14 (10.9)	4 (3.1)	2 (1.6)
*Taenia spp*	-	-	1 (0.8)	-
*S. mansoni*	-	-	1 (0.8)	-
*Giardia lamblilia*	-	28 (21.7)	3 (2.3)	4 (3.1)
*E. histolytica*	1 (0.8)	3 (2.3)	1 (0.8)	3 (2.3)
**Bacteria**				
*Shigella* species	1 (0.8)	4 (3.1)	1 (0.8)	-
*Salmonella* species	5 (3.9)	7 (5.4)	3 (2.3)	1 (0.8)
***Total***	**7 (5.4)**	**75 (58.1)**	**26 (20.2)**	**21 (16.3)**

**Table 3:** Frequency of multiple (double and triple) parasitic infections of children

***Parasite combinations***	***No *%**
**As, Hy, GL**	3 (15.8)
**As, Tt, GL**	1 (5.3)
**As, Tt, EH**	1 (5.3)
**As, HW**	2 (10.5)
**As, Tt**	2 (10.5)
**As, GL**	2 (10.5)
**Hy, GL**	2 (10.5)
**Tt, GL**	3 (15.8)
**GL, Eh**	3 (15.8)
**Total**	19 (100.0)

Keys: - Al- *Ascaris lumbricoide*, Hy: *Hyminolopis nana*, GL: *Giardia lamblia*, Tt: *Trichuris trichiuria*, Eh: *Entamoeba histolytica*, HW: Hook worm.

*Shigella* species showed hundred percent resistances to ampicillin, amoxacillin, and cotrimoxazole while all (100%) isolates were susceptible to ceftriaxone, ciprofloxacin and gentamicin (Table 4). Resistance to ampicillin, cotrimoxazole, chloramphenicol and nalidixic acid was observed in 62.5, 31.3, 18.8% and 12.5 of *Salmonella* isolate respectively. All Salmonella isolates were resistant against Amoxicillin and susceptible to ceftriaxone, ciprofloxacin and gentamicin (Table 4).

**Table 4:** **Resistance pattern of *Shigella *** **and *Salmonella *** **species**

**Antimicrobial**	**No (%) of resistance**	
	***Shigella*** **species (n = 6)**	***Salmonella*** **spices (n = 16)**
Ampicillin	6 (100)	10 (62.5)
Amoxicillin	6 (100)	16 (100)
Cotrimethoxazole	6 (100)	5 (31.3)
Nalidixic acid	1 (16.7)	2 (12.5)
Ceftriaxone	-	-
Ciprofloxacin	-	-
Chloramphenicol	1 (16.7)	3 (18.8)
Gentamicin	-	-

Over all three resistance patterns were seen among *Shigella* isolates. All *Shigella* species were multi-drug resistant (resistant to three or more antimicrobial drugs). About 66.6% of *Shigella* species were resistant to three (ampicillin, amoxacillin, cotrimoxazole) antibiotics. On the other hand 62.5% of *Salmonella* species were multi-drug resistant, ranging from 2 to 4 drugs (Table 5).

**Table 5:** **Antibiogram of *Shigella *** **and *Salmonella *** **isolates**

**Resistance pattern**	**Resistant isolates no. (%)**	
	***Shigella*** **species (N = 6)**	***Salmonella*** **species (*N*** **=** ***16*)**
Amp	-	1 (6.3)
Amx	-	5 (31.3)
Amp, Amx	-	1 (6.3)
Amp, Amx, C	-	1 (6.3)
Amp, Amx, Sxt	4 (66.6)	3 (18.8)
Amp, Amx, NA,	-	2 (12.5)
Amp, Amx, Sxt, C	1 (16.7)	2 (12.5)
Amp, Amx, Sxt, NA,	1 (16.7)	-

Keys:- Amp: Ampicillin, Amx: Amoxicillin, C: Chloramphenicol, NA: Nalidixic acid, Sxt: Cotrimethoxazole.

## Discussion

The study results indicated, of the total 260 study participants, 107 (41.1%) of the symptomatic children were infected with one or more intestinal parasites. It is comparable with study done by Unasho in southern Ethiopia, where 170 (41.9%) children were found to have single and double intestinal parasitic infections [23], but higher than study conducted in Gondar where the observed prevalence of intestinal parasites was 34.2% [24] and the study conducted in Gamo area where 342 (39.9%) study subjects were found positive for at least one intestinal parasite [25]. Our study prevalence result was lower compared with reports of other similar studies, 72.9% in Gondar, Azezo [25], 83% in Jimma [5] and 83.8% in South East of Lake Langano [26]. These variations in prevalence might be due to differences in climatic conditions, environmental sanitation, economic and educational status of parents and study subjects, and previous control efforts. The low prevalence of intestinal parasite in this study compared to the other previous studies in Jimma (5) and elsewhere in the country (7) could be due to increased awareness of the community about personal and environmental hygiene from the continuous awareness creation and interventions made by the health science students from Jimma University during their practical training conducted in the field as well as in different health institutions.

Among helminthiasis, *Ascaris lumbricoid* (11.5%) was the most prevalent parasite and followed by *H. nana* and *T.trichiura*, whereas giardiasis was the leading infection among protozoan infections. Though the rate of prevalence is different the dominancy of *A. lumbricoides* is in line with the study result by Abayne in south Ethiopia [23] and with previous studies done in Assedabo (Jimma, Ethiopia), where *Ascaris lumbricoides* was the leading (56.4%) [5]. But different with study conducted in Gondar [24] where the dominant parasite was *H. nana*. The difference could be mainly difference in climatic condition.

Many studies had already demonstrated the high prevalence of parasitic infections among Ethiopian children in various parts of the country [5,27-29]. In this study, *A. lumbricoides* (3.5%) and *T. trichiura* (3.1%) were found to be the dominant soil transmitted helminthes. Studies done by Ali *et al*. (1999) and Roma and Solomon (1997) reported higher rates of *A. lumbricoides* (54.6% vs. 75.2%) and *T. trichiura* (21% vs. 24.4%) respectively [5,29]. Our study data showed comparable prevalence rate with reported by Tadesse (2005) where the prevalence rate of *A. lumbricoides* and *T. trichiura* were 3.6% and 3.9%, respectively [30]. A prevalence rate of 3% for *H. nana* is higher than earlier findings, which were 1.1% in Wondogenet, Ethiopia [23], 1.3% in south Wollo [31] and lower than with the findings of Tadesse, (2005) and Haileamlak (2005) with frequency rate of 10.1% and 4.3% respectively [27,30].

Except in the early stage/age (where mostly rely on breast feeding), parasitic infections were decreasing as the age of the children increases, which could be due to acquired immunity as they are exposed more and more to infections as well as developing awarness to personal hygiene. In this study multiple parasitic infections were seen in 19 children (7.3%) from the total 260 study subjects and 17.8% among children who were infected with parasites and this result is higher than the study conducted in Gondar, where multiple infections (polyparasitism) occurred in 14 individuals or 4.6% of the total examined subjects and 13.5% of those who had intestinal parasites [32]. The difference could be due to geographical location or variation in study subject sample size.

Comparison with previous study results conducted in different parts of Ethiopia is difficult since the parasite prevalence varies with agro-ecozone, altitude and other environmental factors which are not studied here. Shigellosis is primary a pediatric disease, with more than half of all infections occurring in children between six month to 10 years of age as observed in previous Ethiopian study [14]. The isolation of *Shigella* species (2.3%) in this study is lower than (5%) reported by Mache, 2001 [14] (20.1%) from the same study subjects and area.

Even though, the study was conducted in different age groups, our prevalence rate of 2.3% lowers than that a report by Ashenafi, 1983 (9%) [33] and 11.7% isolation rate reported by Asrat *et al*. 1999 at Tikur Anbessa, Ethio-Swedish children’s hospital [34], a report by Ayalu (6.7%) in Harar [35] and a report 15.6% by Hiruy in Gondar [36]. The low isolation of *Shigella* in this study compared to the previous study in Jimma (14) could be due to increased awareness of the community about personal and environmental hygiene from the continuous interventions made by the health science students form Jimma University during their filed practice.

Epidemiological investigation of salmonellosis in developing countries like Ethiopia is difficult because of the very limited scope of the studies and lack of coordinated surveillance systems. The overall prevalence of *Salmonella* in this study was 6.2%. This is comparable with studies conducted in Ethiopia at different times, 4.5% in Addis Ababa [37], 6.4% in Addis Ababa [16], 4.5% in Addis Ababa [33] and higher than the findings reported by Asrat *et al*. 1999 (3.8%) in Addis Ababa [34] but lower than reported in Jimma (15%) [15]. Antibiotic susceptibility data to *Shigella* isolates showed that all isolates were resistance to ampicillin, amoxacillin, and cotrimoxazole. Similarly a study conducted in Awassa showed that all isolates were resistant to amoxacillin and ampicillin (12). The development of high resistance of *Shigella* species against the commonly used antibiotics was witnessed by other investigators in different periods. In Hawassa high rate of resistance of *Shigella* species to ampicillin (93%), erythromycin (90%), tetracycline (90%) and cotrimoxazole (56%) was reported by Roma *et al*[12], in Gondar, high antibiotic resistance was documented against ampicillin (79.9%), tetracycline (86%), and cotrimoxazole (73.4%) by Yismaw *et al.*[38]. Asrat reported isolation of *Shigella* species with high resistance to erythromycin (100%), Tetracycline (97.3%), and ampicillin (78.7%) in Addis Ababa [39]. High resistance against amoxicillin (100%) and ampicillin (100%) was also reported by Reda *et al.* in Harar [35]. Our Shigella isolates were highly susceptible to ceftriaxone, ciprofloxacin and gentamicin. Comparatively high rate of resistance to ciprofloxacin (8.3%) was reported in Gondar. In parallel to our result lower (2%) resistance rate was reported from Gondar [38]. Comparable to the study conducted by Daniel [39] in Addis Ababa where all *Shigella* isolates were susceptible to gentamicin. The low resistant rate of isolates to chloramphenicol (18.8%), could be that physicians stopped to prescribe the drug before long time a go and once again the strains started to become sensitive. All Shigella isolates were MDR (resistant to two or more drugs). Similar findings were seen in other studies in Ethiopia [12,38].

Infection with non-typhoidal Salmonella in infants and children commonly produces self-limited diarrhoea. Studies have indicated that antimicrobial treatment for uncomplicated gastroenteritis does not shorten the duration and severity of symptoms; in contrast, it may prolong fecal excretion, increase the risk of relapse, and result in the emergence of antibiotic resistance [40]. Nevertheless, if extra-intestinal complications occur, effective antimicrobial treatment is essential. Multidrug resistant phenotypes have been increasingly described among *Salmonella* species worldwide, according to the infectious disease report released by the WHO in 2000 [41].

In this study, *Salmonella* isolates showed high re resistant to ampicillin and amoxicillin which is comparable with previous study done in Harar, Ethiopia where the highest level of resistance was detected to ampicillin (100%) and amoxicillin (100%) [35]. a relatively similar pattern of resistance (74 to 97.3%) was reported from other parts of the country [42] and outside [43]. Our study findings showed that all *Salmonella* species were susceptible to ceftriaxone and ciprofloxacin, which is inline with recently study results conducted in Nigeria where all isolates were susceptible to ciprofloxacin, and ceftriaxone [44]. Un like most previous study findings, in this study *Shigella* and *Salmonella* species showed low resistance level to chloramphenicol, this could be due abandoning of prescribing the drug by the responsible health personnel before a long time ago.

## Conclusion

This study indicated that intestinal parasite and some enteric bacteria such as *Salmonella* and *Shigella* species are responsible for the majority cases of diarrhoel in children. The results of the present study suggested that antibiotics which were more commonly prescribed in the study area previously, like ampicillin, amoxicillin and cotrimethoxazole developed resistance to *Shigella* and *Salmonella* species and should not be used as empirical treatment of diarrhoea in children at least in the study area. Therefore, measures including health education, improvement of safe water supply, sanitation facilities and continuous monitoring of microbiological and antimicrobial surveillance is crucial.

## Competing interests

The authors declared that they have no competing interests.

## Authors’ contribution

GB: Participated from inception of the research question to design, analysis, interpretation and preparation of the manuscript. HT: - Participated in proposal development, analyzed the data, edit and wrote the manuscript for publication. Both authors have given final approval of the version to be published.
